# Antiviral activity of triptolide on herpes simplex virus in vitro

**DOI:** 10.1002/iid3.667

**Published:** 2022-06-20

**Authors:** Nasrin Aliabadi, Marzieh Jamalidoust, Gholamreza Pouladfar, Atoosa Ziyaeyan, Mazyar Ziyaeyan

**Affiliations:** ^1^ Department of Clinical Virology, Clinical Microbiology Research Center, Namazi Hospital Shiraz University of Medical Sciences Shiraz Iran; ^2^ Osteoarthritis Research Program, Division of Orthopedic Surgery, Schroder Arthritis Institute University Health Network Toronto Canada

**Keywords:** antiviral, HSV‐1, ICP4, natural product, triptolide

## Abstract

**Background:**

Herpes simplex virus‐type 1 (HSV‐1) can cause diseases, especially amongst neonates and immunocompromised hosts. Hence, developing a novel anti‐HSV‐1 drug with low‐level toxicity is vital. Triptolide (TP), a diterpenoid triepoxide is a natural product with range of bioactivity qualities.

**Methods:**

In this study, viral infection was assessed in different phases of the HSV‐1 replication cycle on A549 cells, using various assays, such as adsorption inhibition assay, penetration inhibition assay, time‐of‐addition assay, and quantitative polymerase chain reaction (qPCR).

**Results:**

The results indicate that TP can effectively inhibit HSV‐1 infection in the lowest range of concentration. TP exhibited significant inhibitory effect on HSV‐1 plaque formation, with 50% effective concentration (EC50) of 0.05 µM. Furthermore, the time‐of‐addition assay suggests that TP has viral inhibitory effects when it was added less than 8 h postinfection (h.p.i.). This result is further confirmed by decline in the expression viral immediate‐early genes (ICP4, ICP22, and ICP27) in 6 h.p.i in the TP‐treated group compared to the control group, evaluated by real‐time qPCR. The Western blotting result was also consistent with the previous findings, which confirms that TP can positively affect ICP4 during HSV‐1 infection.

**Conclusions:**

The TP also showed antiviral activity against HSV‐1. This dose‐dependent activity is an indication of a particular cellular component, rather than cytotoxicity that has mediated its function. Finally, the result suggest a new approach for an effective treatment option of the HSV‐1 infections.

## Graphical abstract

Herpes simplex virus‐type 1 (HSV‐1) causes significant human diseases, especially in neonates and immunocompromised hosts. Thus, developing novel anti‐HSV‐1 drugs with low toxicity is necessary for public health. Triptolide (TP) is a diterpenoid triepoxide that, as a natural product, exhibits a broad range of bioactivities. TP also showed antiviral activity against HSV‐1. This dose‐dependent activity might indicate that a particular cellular component, rather than cytotoxicity, mediated its function. In conclusion, this report proposed new outlooks in investigating the effective treatment options for HSV‐1 infections

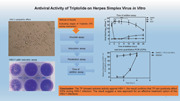

## 1 INTRODUCTION

Herpes simplex virus (HSV), a member of Herpesviridae is an enveloped double‐stranded DNA virus with a linear 152‐kbp nucleic acid.^1^ The HSV genome comprises three major groups of designated immediate early (IE), early (E), and late (L) genes.^1^, ^2^ This virus can create a lifetime latent infection in the neuronal cells. In the neurons, the virus often remains latent, until it is re‐stimulated into a productive cycle

As a global health concern, HSV infections are identified in approximately 50%–90% of individuals with seropositive for HSV‐1.^3^, ^4^ The infection appears with different manifestations, ranging from mild to severe, such as gingivostomatitis, kerato‐conjunctivitis, and herpes encephalitis.^5^ Hence, a novel antiviral drug is necessary to reduce the risk of resistant acyclovir (ACV) development in severe HSV infections, such as immunocompromised transplant recipients and HIV‐infected individuals.^6^ Synthetic nucleoside analogs, such as ACV, valacyclovir, and famciclovir are prescribed as antiherpes compounds for the treatment of various HSV infections. Nonetheless, continual clinical usage of the aforementioned compounds can develop into drug‐resistant.^7^, ^8^ The rate of ACV resistance is usually low among the immunocompetent patients; however, the rate of resistance has significantly increased to 14% from 5% in bone marrow transplant recipients.^9^, ^10^ The rise of ACV‐resistant strains demands prolonged and challenging treatment, with expected relapses and treatment failure.^11^ Therefore, the transmission of resistant strains to the susceptible individuals should be taken seriously.

This calls for a new and effective antiviral agent to either replace or complement the conventional antiherpes drugs.^12^, ^13^ In this regard, alternative antiviral medications have been suggested as an effective therapy models with reduced chance of ACV resistance. Natural products are important sources of molecules that can be purified to become an active components, such as plants or marine‐sourced biological derivatives.^13^, ^14^, ^15^ These components are a valuable source, and many of contemporary drugs and medications are the derivatives of such sources.^16^ Therefore, new antiviral drugs with advanced antiviral actions, antiviral targets, and antiviral mechanisms are essential.^9^, ^17^

The present study aims to investigate the potential anti‐HSV activity of TP as a natural product while considering the virus titer and plaque counts. Additionally, we attempt to provide an insight into the mechanism of action for the compound against HSV‐1 and to present the inhibitory viral replication steps.

## 2 MATERIALS AND METHODS

### 2.1 Cells and virus

A549 and Vero cells were purchased from the Pasteur Institute of Iran. Primary rabbit kidney (PRK) cells were provided by the Center of Comparative and Experimental Medicine in Iran. A549 and Vero cells were cultured in Dulbecco's Modified Eagle's Medium (DMEM) (Gibco), comprising 1% penicillin‐streptomycin (Gibco™, 10,000 U/ml) supplemented with 10% fetal bovine serum (Gibco) at 37°C in 5% CO_2_. The PRK cells were cultured in DMEM (Gibco), containing 1% penicillin‐streptomycin (Gibco™, 10,000 U/ml) and supplemented with 15% fetal bovine serum (Gibco) at 37°C in 5% CO_2_. In this study, the used HSV was described in a previous study.^17^ This virus was propagated in Vero cells and titrated using the endpoint dilution method.^18^ Finally, the cells were stored in aliquots at −70°C. Generally, the ACV assay (0.01 mg/ml) served as the gold standard treatment to inhibit the virus propagation.

### 2.2 Cytotoxicity assay

The in vitro cytotoxicity of the TP on A549 as well as the primary cells was evaluated, using the WST‐1 assay Roche Diagnostics GmbH. Briefly, 5 × 10^3^ A549 and PRK cells were added to a 96‐well plate and then incubated in the TP concentration at 37°C. As the manufacturer's protocol, after 24 h, WST‐1 reagent was performed. The sample absorbance was read at 440 nm (610 nm was used as reference wavelength and then subtracted) in a multi‐plate reader (BioTek™ Epoch™ Microplate Spectrophotometer—Fisher Scientific). Next, the TP's 50% Cytotoxic Concentration (CC50) on A549 and PRK cells was computed. As described previously, HSV‐1 yield reduction assay as well as virus yield reduction assay was performed with minor modifications.^19^ Briefly, A549 were seeded into 24‐well tissue culture plates at a TP in concentrations of (0.01, 0.05, 0.1, 0.2, 0.5 µM), and then incubated at 37°C for 2 h to allow the cells adapt to the new environment. After that, 25 µl diluted virus with 0.1 multiplicity of infection (MOI) was inoculated into the wells. The final assay volume in each well was 500 µl. The plates were then incubated at 37°C for 48 h. Following that, the plates were kept at −70°C with three freezing and thawing cycles and the yields of progeny virus were determined via plaque assay on Vero cells.

Finally, the average plaque counts from the TP wells were calculated and the EC50 values were computed.

## 3 EVALUATION OF ACTIVITY MECHANISM

### 3.1 Virucidal assay

As described previously, the virucidal assay was done.^20^ Briefly, 50 µl of the HSV‐1AN95 stock was mixed with 6 × EC50 µM of TP and 450 µl of the culture medium (without virus as the negative control), and incubated at room temperature (25°C) for 1 h. Finally, the residual from the treated virus suspensions were investigated via titration.

### 3.2 Adsorption assay

As described previously, an adsorption inhibition test was performed to determine the effects of TP on viral attachment.^18^ Briefly, the confluent A549 cell monolayers in 12‐well plates were precooled at 4°C for 1 h. After that, noncytotoxic concentrations of TP (0.05, 0.15, 0.25, and 0.75 µM) and 0.15% DMSO as control were added in triplicate. Immediately after, 200 plaque‐forming units (PFUs)/well of HSV‐1 were added to each well. All the plates were then shifted at 4°C for 3 h to allow viral adsorption. Then, the medium was removed and cells were washed three times with sterile phosphate‐buffered saline (PBS) to remove the nonattached viruses. Finally, 2% agar overlay was added to the plates and incubated at 37°C in a humidified atmosphere comprising of 5% CO_2_ for 48 h until viral analysis plaques were completed.

### 3.3 Penetration assay

As previously reported, the antiviral effect of TP on HSV host cells was evaluated, using plaque reduction method.^21^ Briefly, the 70% confluent A549 cell monolayers in 12‐well plates were pre‐cooled at 4°C for 1 h. Then, 200 PFUs of HSV‐1 were inoculated, and then the plates were incubated at 4°C for 1 h for the completion of virus uptake. Afterward, the cells were washed, using cooled PBS and treated with four different concentrations of TP (0.05, 0.15, 0.25, and 0.75 µM) and 0.15% DMSO. The plates were incubated at 4°C for 30 min, and then immediately after at 37°C to stimulate viral penetration in the presence of the test concentrations. The virus penetration was inhibited during adsorption and treatment, using the following steps at 4°C. After the incubation period of 30 min at 37°C, the supernatants were removed. The cells were treated with (135 mM NaCl, 10 mM KCl, 40 mM Na‐citrate, pH = 3.0) under acidic conditions (low pH) for 45 s to stop the penetration and to disable the attached nonpenetrated viruses. The citrate buffer (low pH) was discarded by washing it three times with PBS, and then the overlay medium was added. As described for the adsorption inhibition assays, the final phase of the experiment was done.

### 3.4 Time of addition assay

As previously described, the antiviral efficiency of TP (3 × EC50% µM) against HSV‐1 was evaluated by adding it to the cultured cells at various points during incubation.^20^ The experiment was carried out in 24‐well plates and each 24‐well plate was inoculated with 200 PFUs of the virus (HSV‐1). The compounds were added 1 h before being infected, immediately after, and at 1, 2, 4, 6, 12, 18, and 24 h postinfection (h.p.i.). The 3 × EC50% µM concentration of the compound and 0.15% DMSO were re‐added to the wells at the specified times after being infected for 48 h. Afterward, Vero cells (5 × 10^5^ cells in 100 ml of DMEM) were seeded in each well of a six‐well plate (SPL, Life Sciences) and then incubated for the plaque assay at 37°C with 5% CO_2_ for 48 h. Finally, the virus infectious titers residual were further assessed.

### 3.5 Quantitative polymerase chain reaction (qPCR)

The infected A549 cells with 200 PFUs of the HSV‐1 were collected at different time points (immediately after infection and 1, 2, 4, 6, 12, 18, and 24 h.p.i) by performing freeze and thaw cycles after the cells were lysed. Then, the viral DNA was extracted, using a High Pure Viral Nucleic Acid Kit (Roche Diagnostics, Basel, Switzerland). The viral DNA was quantified, using real‐time PCR by utilizing an Applied Biosystem step one plus real‐time PCR machine (Applied Biosystem). For HSV‐1, the oligonucleotide primers and probes were used, as previously described in another study.^22^ The HSV DNA was amplified in a 20.0 μl reaction, using qPCRBIO Probe Mix Hi–ROX (PCR Biosystems). The reactions contained 10 μL of 2X qPCRBIO Probe Mix, 0.8 μl primers, 0.8 μl of the probe as well as 5 μl of the extracted DNA sample. Within 2 min of polymerase activation at 95°C, the PCR mixture was subjected to 45 PCR cycles at 95°C for 5 s and then at 60°C for 20 s.

### 3.6 Reverse‐transcriptase qPCR

The A549 cells were infected with HSV‐1 (MOI of 0.1) and treated with TP and ACV (3 × EC50% µM) immediately after infection and 1, 2, 4, 6, 12, 18, and 24 h.p.i. The viral RNA was extracted from the samples using a High Pure Viral RNA Kit (Roche Diagnostics). The purified total RNAs were then tested according to a previously published study^23^ to quantify the IE genes (α4, US1, and UL54 [ICP4, ICP22, and ICP27]), the E gene (UL29 [ICP8]), and (L) genes (UL36, UL48, UL27, UL22, and US6 [glycoprotein gB, gH, gD, VP1/2, and VP16]). These genes were evaluated, using qPCRBIO SyGreen One‐Step Hi‐ROX‐based real‐time quantitative PCR (Catalogue number: PB25.12, PCR Biosystems) by means of an Applied Biosystem step one plus real‐time PCR machine (Applied Biosystem). Real‐time quantitative PCR was performed in triplicate followed by the two following steps: 95°C for 2 min and 45 cycles at 95°C for 5 s, at 60°C for 10 s, and 72°C for 15 s. Reverse‐Transcriptase quantitative PCR (RT‐qPCR) and the used primers are described in Table 1.^23^ As the endogenous control, the GAPDH gene was used to normalize the differences in total RNA of each sample. The analysis was done, using the $2^{‐∆∆C_{t}}$ threshold cycle.

**Table 1:** Primers used for quantitative real‐time polymerase chain reaction

Gene	Primer name		Sequence (5′→3′)
*U*_*L*_ *27*	gB	Forward	AAACCGAAAAACCCACCGCC
		Reverse	TGTTCTCCGCCTTGATGTCC
*U*_*S*_ *6*	gD	Forward	GCCCCGCTGGAACTACTATG
		Reverse	TTATCTTCACGAGCCGCAGG
*U*_*L*_ *22*	gH	Forward	GGTTTATGGTTCGTGGGGGT
		Reverse	CTGTCTGCTCAGTCCAGTCG
*U*_*L*_ *36*	VP1/2	Forward	CGGGTCAAAAAGGTATGCGG
		Reverse	TGTCGTACACGCTCCTAACC
*U*_*L*_ *48*	VP16	Forward	TTTGACCCGCGAGATCCTAT
		Reverse	GCTCCGTTGACGAACATGAA
*α4*	ICP4	Forward	CGACACGGATCCACGACCC
		Reverse	GATCCCCCTCCCGCGCTTCGTCCG
*U*_*L*_ *29*	ICP8	Forward	CGACAGTAACGCCAGAAG
		Reverse	GGAGACAAAGCCCAAGAC
*U*_*L*_ *54*	ICP27	Forward	ATGTGCATCCACCACAACCT
		Reverse	TCCTTAATGTCCGCCAGACG
*U*_*S*_ *1*	ICP22	Forward	CGCCGCAGAAGACCGCAAGT
		Reverse	TGTCGCTGCACGGATAGGG
GAPDH	GAPDH	Forward	GGTGGTCTCCTCTGACTTCAACA
		Reverse	GTTGCTGTAGCCAAATTCGTTGT

### 3.7 SDS–PAGE and immunoblotting assays

The effects of TP on the yields of three viral proteins including HSV‐1 (gD [US6] gene), ICP4 (*α4*), and ICP0 (*α0*) were examined. SDS–PAGE was used to analyze the samples (15 µl each), after adding 10% polyacrylamide gel run at 150 V (MiniProtean 3; Bio‐Rad), which was then transferred to a transfer buffer (0.248 M Tris–HCl pH 8.8 1.92 M glycine, 20% methanol). Next, the nitrocellulose membrane was added to the bottom of the gel. Then, the gel was run at 60 V for 3 h to obtain the proteins. The binding sites that were nonspecific on the membranes were blocked with 5% skim milk dissolved in Tris buffer saline, containing 0.1% Tween‐20 (TBST) at ambient temperature for 50 min. The blots were incubated with appropriate antibodies of H1A027 and HA021. Anti‐HSV‐1 ICP0, ICP4 monoclonal antibodies (Ref. MAB‐13583, MAB‐13584 Abnova) diluted 1:2000 and anti‐HSV‐1/2 gD monoclonal antibody (Ref. MAB‐13574, Abnova) diluted 1:2000 were used. The anti‐GAPDH monoclonal antibody (H00002597‐M01‐ Abnova) was incubated overnight at 4°C. After three washes with TBST, the blots were incubated, using Goat Anti‐Mouse IgG (H&L), secondary antibody (Peroxidase), or mouse anti‐mouse secondary antibodies (PAB0096, Abnova, Canada) (1:1000) at room temperature for 1 h. Finally, the blots were washed three times with TBST. The membranes were incubated with horseradish peroxidase‐conjugated secondary antibodies at room temperature for 2 h. The bands intensities were calculated, using GeneTools SyneGene Software for Windows.

### 3.8 Statistical analysis

The selectivity indices (SI, CC50/EC50) of each test, dose—inhibitory rate curve, and regression analysis of the dose—viability curve were calculated, using GraphPad Prism 5 (GraphPad Software Inc.). Additionally, the one‐way analysis of variance (ANOVA) was used to compare the treated groups. The *p* < .05 was considered to be statistically significant.

## 4 RESULTS

### 4.1 Cytotoxicity of TP on A549 and PRK cells

The PRK and A549 cells were treated with TP (at concentrations of 0.5, 1.0, 1.5, 2.0, 4.0, and 8.0 μM) to assess their cytotoxicity. After 48 h of incubation, cell viability was evaluated, using the WST‐1 assay. The CC50 of TP was 4.454 μM on the A549 cells and 21.46 μM on the PRK cells (Table 2).

**Table 2:** Antiviral activity (EC50) on herpes simplex virus type 1 (HSV‐1), cytotoxicity (CC50), and selectivity index (SI) data for triptolide on A549 cells and Primary cells.

Compound	CC50 (µM)	EC50 (µM)^c^	SI (CC50/EC50)
Triptolide	4.454^a^	0.05^c^	89.08
Triptolide	21.46^b^	0.065^c^	330.15

^a^ Values obtained by WST‐1 on A549 cell.

^b^ Values obtained by WST‐1 on primary cell.

^c^ Values obtained by virus yield reduction assay.

### 4.2 Plaque reduction assays and revealing the half‐maximal effective concentrations

The antiviral efficacy of TP in HSV‐1 infectivity was initially assessed, using virus yield reduction assay that was performed on A549 and PRK cells in vitro. A five‐point dose‐response analysis was then performed in triplicate in two secondary assays including the same cytopathic effect (CPE) assay done during the primary screening as well as a parallel EC50 assay. The results are presented as percentage of the detected plaques reduction in the group treated with the natural product in comparison with the DMSO group. The plaques were scanned and counted on the Vero cells. The results revealed that TP can inhibit HSV‐1 with an EC50 value of 0.05 μM (Table 2). To assess the SI of the compound, CC50 values were determined, using the WST‐1 assay. Accordingly, the SI for the TP was 89.08 on A549, and the SI was 330.15 on the PRK cells (Table 2), using regression analysis (dose–viability curve).

### 4.3 Evaluation of the possible antiviral mechanism

**Figure 1:**
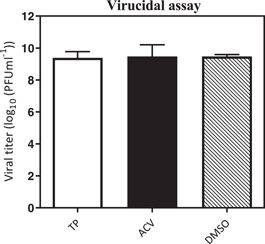
The mechanism of the triptolide (TP)‐mediated inhibition of herpes simplex virus‐type 1 (HSV‐1). Direct virucidal effect test. HSV‐1 was mixed with TP (6 × EC50% µM), acyclovir (ACV) (6 ×  EC50% µM), or DMSO (0.15%%) (consisting of DMSO, virus, and cells) and was then incubated at room temperature (25°C) for 1 h. The residual infectious virus titers were demonstrated by the plaque assay. The data were obtained from three independent experiments. Error bars indicate standard deviations from the three independent experiments.

**Figure 2:**
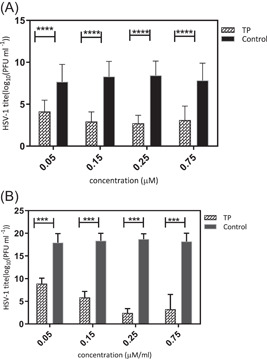
(A) The effect of triptolide (TP) on virus adsorption and (B) penetration. This procedure was performed as described in Section 2. The data represent the results of three independent experiments that were performed repeatedly. Statistical significance between the compound‐treated and DMSO‐treated groups was determined by the Student's *t*‐test: ns; ***p < .001; ****p < .0001.

The infected HSV‐1 particles were incubated along with the TP before monolayer infection to see whether the selected TP could directly deactivate the virus particles. According to the results in Figure 1, this compound did not have any virucidal properties. The antiviral activity of TP was also assessed when entering cells or interference during the initial phase of the viral replication including, the adsorption and penetration phase of the virus in the Vero cells. As for the adsorption inhibition experiments, the cell monolayers were pre‐incubated with HSV‐1 in the presence of TP at 4°C. The results showed a significant reduction in the virus‐induced lysis plaques count, which was detected after adsorption of TP compared to DMSO (Figure 2A).

During penetration phase, cells were incubated at 4°C for 30 min, and then they were placed in an environment at 37°C to stimulate the virus uptake in the presence of this compound. After that, the virus yield decreased significantly compared to DMSO (Figure 2B). Furthermore, the adsorption and penetration assay results revealed that TP can directly kill or block the HSV‐1 when attempting to enter the A549 cells.

**Figure 3:**
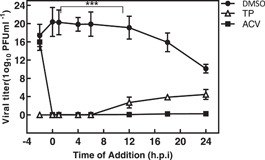
The time‐of‐addition assay. A549 cells were infected with herpes simplex virus‐type 1 (HSV‐1) at the MOI of 0.1. Then, triptolide (TP) (3 × EC50% µM), ACV (0.01 μg/ml), and 0.15% DMSO were added to the cells at the indicated time points both before and after infection. The infected cells were lysed by performing three cycles of freezing and thawing. The residual infectivity of the treated viruses in different time courses was further investigated by titration on the Vero cells. The values were obtained by the virus yield titration of cell lysates and represented the mean of an independent experiment in triplicate (±SE). *** indicates significant differences between the tested sample and DMSO (*p* < .0001). ANOVA/Dunnett's tests were carried out, as appropriate. ACV, acyclovir.

The time‐of‐addition assay was performed to identify the probable phase (s) of virus replication targeted by the TP. The residual infectivity of the treated virus in different time period was further investigated by titration on the Vero cells. As shown in Figure 3, the virus yield titration was high, but significantly decreased when TP was introduced before 8 h.p.i. The virus level yield titration in the ACV‐treated cultures was nearly the same as those obtained at baseline.

### 4.4 Viral DNA yield in the presence of TP

**Figure 4:**
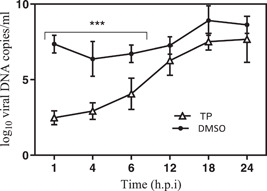
Quantitative polymerase chain reaction was performed to assess the number of herpes simplex virus‐type 1 (HSV‐1) genome copies after treatment with triptolide (TP) (a) in the presence or absence of these compounds compared to the DMSO control (consisting of the virus, 0.15% DMSO, and cells). The data have been presented as mean and standard deviation. Significant differences were found between the TP and DMSO control groups (consisting of the virus, 0.15% DMSO, and cells) regarding the HSV‐1 DNA level up to 8 h.p.i. *** indicates significant differences between the tested sample and the virus control (*p* < .0001). ANOVA/Dunnett's tests were carried out, as appropriate.

Random examinations were carried out in the presence or absence of compound when examining the antiviral effect of TP on HSV‐1 DNA yields, 1 h before infection, immediately after infection, and 1, 2, 4, 8, 14, and 24 h.p.i. Analyses showed that the TP significantly inhibited HSV‐1 DNA synthesis if it was introduced up to 8 h.p.i. (Figure 4).

### 4.5 The effect of TP on the expression of HSV‐1 immediate‐early, early, and late genes

**Figure 5:**
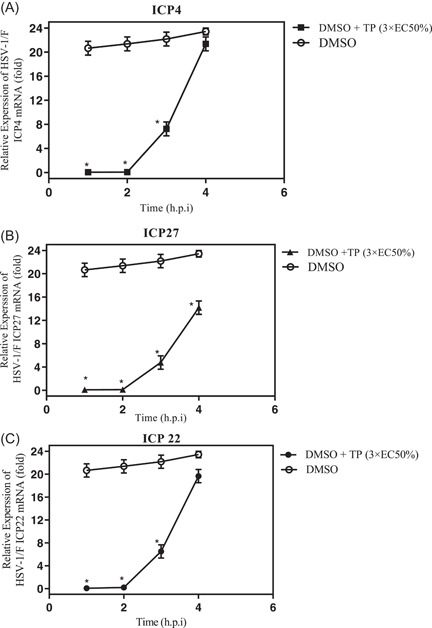
The effect of triptolide (TP) (3 × EC50% µM) on the expression of herpes simplex virus‐type 1 (HSV‐1) (immediate‐early genes [IE]) (A)–(C). A549 cells were infected with HSV‐1 at the MOI of 0.1 and were then treated with and without 3 × EC50% µM of the TP compound. RNA was extracted at specified intervals and real‐time quantitative polymerase chain reaction was done using mRNA‐specific primers. Gene transcription level was defined as relative based on the mRNA fold change ($2^{‐∆∆C_{t}}$) at each time point. It was initially normalized by GAPDH and was then compared to the DMSO group (consisting of the virus, 0.15% DMSO, and cells) at 4 h.p.i. ANOVA/Dunnett's tests were carried out, as appropriate.

The mRNA levels in the three groups of HSV‐1 genes (IE, E, and L) were evaluated through the normalized reference gene (GAPDH), using the $2^{‐∆∆C_{t}}$ method for the relative quantification RT‐qPCR to determine the efficacy of the compound in the HSV replication cycle. The relative mRNA levels of IE at 4 h.p.i. are shown in Figure 5A–C. Accordingly, the mRNA levels of the ICP4, ICP22, and ICP27 genes were relatively low in the TP‐treated group up to 4 h.p.i. However, their mRNA levels increased in the DMSO group at 4 h.p.i. Further, the expression patterns of E and L genes (glycoprotein gB, gH, gD VP1/2, VP16, and ICP8) in the presence of the TP were similar to those of the DMSO group at 12 h.p.i. (data not shown). As the IE regulatory proteins are necessary for transition of IE transcription to later viral gene transcription, ICP4, ICP22, and ICP27 genes had the greatest effect on the early and late promoters. Moreover, gD acts as an L protein, which is an envelope glycoprotein, binding the potential host cell entry receptors. This glycoprotein stimulates the viral fusion through the host membrane by employing the fusion mechanism that comprise of gB and gH/gL.^1^

**Figure 6:**
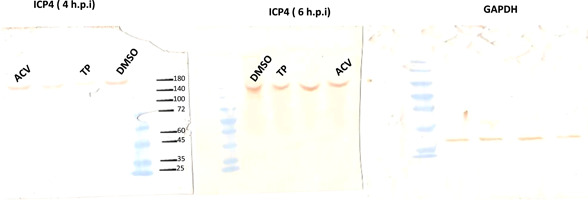
For western blot analysis of ICP4, the cells grown in a six‐well plate were infected with HSV‐1 at the MOI of 0.01 and were treated with or without 3 × EC50 μM TP. Then, the cells were harvested. There was a noticeable difference between the 3 × EC50 μM TP and 0.15% DMSO groups at 4 and 6 h.p.i. Molecular weight standards in kiloDaltons are indicated to the left of each panel.

The results were validated through Western blot analysis. Accordingly, TP was tested for its antiviral properties to see, if it can inhibit the ICP4 and gD genes expression. Additionally, the two compound‐treated cells were compared with the viral controls, to see whether they can reduce the ICP4 or gD proteins. To be noted, the assay sensitivity as well as the MOI values were the same in western blot as the RT‐qPCR assays, which were described in Section 2. Blotting showed that the ICP4 protein levels in TP at 4 h.p.i., were not close to the ACV and DMSO groups, which was in line with the RT‐qPCR results concerning TP (Figure 6).

## 5 DISCUSSION

The problem of drug‐resistant strains has become a global concern. Hence, new strategies are warranted to eradicate HSV infections amongst humans.^24^ Given the high rate of HSV infections, the high prevalence is not expected to be eliminated in the near future.^10^ The rise in number of immunocompromised patients as well as the treatment time might further magnify the issues that are deep rooted in drug‐resistant HSVs.^9^ Despite the effectiveness of standard treatments and low rate of drug resistance, novel treatments must be developed to prevent possible HSV epidemics.^11^ According to the literature, when comparing natural agents with synthetic ones, they proved to be more efficient, especially antimicrobial and anticancer agents.^13^, ^25^, ^26^ So far, many studies were carried out on products, such as crude extracts, fractions, and pure compounds that were either isolated from plants, animals, microorganisms, or marine life for their antiviral effect on HSV.^14^ Therefore, the anti‐HSV agents properties have to be assessed as an alternative to nucleoside analogs for more effective treatment.^15^

From 1981 to 2010, about 34% of the natural product byproducts, which were approved by the Food and Drug Administration (FDA) were either natural products or their direct spin‐offs.^14^ To identify the new inhibitors of HSV‐1, natural product library, consisting of 133 compounds was initially screened. In a previous study, TP was identified as the inhibitor of HSV‐1 infections, using a high‐throughput screening assay. The hit rate of inhibition (60% CPE inhibition) was greater than any previous screening of the FDA‐approved drug libraries (0.23% with a lower cut‐off of around 30% CPE inhibition).^27^

TP is a diterpenoid triepoxide, which is a purified roots of the Chinese herb, *Tripterygium wilfordii*. This natural product has a broad range of bioactivities with anti‐inflammatory, antitumor, immunosuppressive, and antimicrobial effects.^28^ The antiviral activity of TP was reported on HIV‐1 and Kaposi's Sarcoma‐associated Herpesvirus (KSHV). TP was also found to inhibit HIV‐1 replication by promoting the proteasomal degradation of the Tat protein.^29^, ^30^ Triptofordin C‐2 isolated from the *T. wilfordii* Hook was also found to be active against HSV‐1 and Human Cytomegalovirus (HCMV).^31^ However, the antiviral effect of TP on HSV‐1 is still unknown.

In the present study, to confirm the TP effects on virus replication, we exploited the PRK and A549 cells. The A549 cell has a human origin. The reason for that was to study the human cell responses to the TP as well as virus replication. In the next step, we used PRK cell, which is highly sensitive to HSV virus. The Cytotoxicity assay result showed that the TP anticancer properties have no effect on cell proliferation and that the TP was capable of preventing the virus from replication. Similarly, TP exhibited significant inhibitory effect with the EC50 of 0.05 µM on the A549 cells. Additionally, TP was highly effective in reducing the HSV‐1 titration on the PRK and A549 cells. According to different studies results that had examined the TP toxicity, IC50 value was between 0.2 and 0.346 µM on HepG2 and NRK‐52E cells.^32^, ^33^ Accordingly, the highest concentration used in our assays, was 0.05 µM, which did not exhibit any cytotoxicity effect against PRK and A549 cell based on the WST‐1 assay. In addition, the EC50 of ACV was 0.01 μg/ml concentration on the A549 cells, when applied as the gold standard antiviral. Notably, the HSV‐1 isolate in this study was sensitive to ACV, which was in line with a previous study.^34^ Moreover, the WST‐1 assay was used to calculate the SI of the TP, which revealed the CC50 of 89.08 µM (Table 2). Tsuchiya et al. stated that SI values >1 exhibited antiviral activity.^17^ However, due to differences between the cytotoxic and antiviral concentrations, greater SI values are warranted to recommend a safe antiviral therapy margin. In the present study, the antiviral activity of TP was less than the cytotoxic concentrations in a dose‐dependent manner, indicating that it might be mediated by a particular mechanism rather than cytotoxicity.

Consequently, viral infectivity was assessed on the A549 cells in different HSV‐1 replication cycles in a time‐dependent manner. The results of virus yield reduction assays validated that TP was effective during IE and L stages within 4‐8 h of viral replication. Besides, TP had an inhibitory effect during the virus entry sites; hence, complementary assays that targeted the early replication steps were carried out. The study findings related to virus inactivation, adsorption, and penetration assays indicated that TP can affect viral adsorption and entry into cells at the MOI of 0.01 PFU/cell. Therefore, TP exhibited remarkable antiviral effect on HSV‐1 infection via inhibiting viral plaque formation and new virus production. The results also revealed a significant reduction in the HSV‐1 virus titers in the presence of TP. In this study, virus inhibition uptake can be associated with the direct inactivation of viruses or changes in the receptors on the cell surface, which is vital for virus entry into the cells by the treated compound. This effect was noticeable amongst the tested natural products.

In this study, a virucidal experiment was conducted to determine the deactivating ability of TP on virions, but the results showed no inhibitory effect on the virus compared to DMSO. It is generally believed that a good antiviral agent should be able to reduce the virus infection by 2 log_10_ (99% inactive) or more.^35^ Despite the adsorption inhibition effects, TP required a concentration‐dependent inhibitory activity on either virus entry or uncoating. Complete virus entry arrest was observed at 3 × EC50% μM concentration (Figures 2A,B). M. Micaeal Gonzalez et al. found that the β‐carbolines were not virucidal, and did not cause any disturbances for the attachment or penetration of HSV‐1. Min‐ke Li also reported that arbidol hydrochloride was not able to inhibit HSV‐1 from attaching to HeLa and Vero cell surfaces (MOI = 0.0001 PFU/cell). Similarly, other studies indicated that compounds^36^ could not inhibit HSV‐1 during adsorption and penetration stages. Interestingly, the results of the time‐of‐addition assay showed that the critical time for the inhibitory effect of TP on HSV‐1 replication was about 8 h.p.i; where, the viral DNA replication site, L gene transcription, and viral DNA encapsidation took place at the HSV replication compartments.^37^ On the other hand, TP had inhibitory effects on HSV‐DNA synthesis. The ACV as gold standard revealed viral DNA levels similar to the DNA concentrations during the inoculation period (Figure 4).

In the present study, a kinetic study was performed, using real‐time PCR to evaluate the effect of TP on viral DNA synthesis. The results revealed reduction in the DNA concentration of the cultures that contained TP in comparison to DMSO. Since the measured DNA over 24 h corresponds with the genomic DNA of the new viruses, the finding implies that the inhibitory activity of this compound might have been in the replication stage, which could interfere with the production of the viral progeny by releasing progeny virions from the host cells during RNA or protein synthesis, maturation, encapsulation, and/or virus spread. This is consistent with the IE and E inhibition that was detected in the time‐of‐addition assay. Finally, TP can inhibit the viral genes expression in the IE stage. Hence, HSV‐1 was actively replicated in the nucleus by entering the host cells. Generally, the findings suggest that TP can affect some cell‐independent viral processes by selective cessation by ultimately interacting with a viral transcription factor that inhibits the synthesis of viral mRNAs that can lead to the restricted production of viral proteins. Due to limited data on antiviral effects of these compounds, further studies with focus on the molecular mechanisms of anti‐HSV‐1 activity in vivo are warranted.

## AUTHOR CONTRIBUTIONS

Nasrin Aliabadi contributed to study concept, data search, data extraction and analysis, and drafting of the manuscript; Marzieh Jamaliduost contributed to data extraction and analysis, critical revision of the manuscript; Gholamreza Pouladfar contributed to data extraction and drafting of the manuscript; Atoosa Ziyaeyan contributed to data extraction and drafting of the manuscript; Mazyar Ziyaeyan contributed to study concept, supervision, revision of the manuscript, guarantor of the article. All authors read and approved the final manuscript before submission.

## CONFLICT OF INTEREST

The authors declare no conflict of interest.

## ETHICS STATEMENT

The study design and protocols were ethically approved by the Department of Medical Ethics and Philosophy of Health, Shiraz University of Medical Sciences, Iran under IR.SUMS.REC.1397.901. The written informed consent was taken from the participant we took his sample.
